# Oxytocin administration versus emotion training in healthy males: considerations for future research

**DOI:** 10.1098/rstb.2021.0056

**Published:** 2022-08-29

**Authors:** Katie Daughters, D. Aled Rees, Laura Hunnikin, Amy Wells, Jeremy Hall, Stephanie van Goozen

**Affiliations:** ^1^ Neuroscience and Mental Health Research Institute, Cardiff University, Cardiff, UK; ^2^ Centre for Human Developmental Science, Cardiff University, Cardiff, UK

**Keywords:** oxytocin, emotion training, emotion recognition, empathy

## Abstract

Identifying emotions correctly is essential for successful social interaction. There is therefore a keen interest in designing therapeutic interventions to improve emotion recognition in individuals who struggle with social interaction. The neuropeptide oxytocin has been proposed as a potential physiological intervention due to its important role in emotion recognition and other aspects of social cognition. However, there are a number of caveats to consider with the current form of intranasal oxytocin commonly used in the literature. Psychological interventions, on the other hand, do not carry the same caveats, and there is, therefore, a need to understand how intranasal oxytocin administration compares to psychological interventions designed to target the same psychological phenomena; and whether a combined intervention approach may provide additive benefits. Here we present a pilot, proof-of-concept study in healthy volunteers comparing the effect of intranasal oxytocin against a validated emotion training programme, finding that the psychological intervention, and not intranasal oxytocin, improved emotion recognition specifically for angry expressions. We discuss the theoretical implications of the research for future clinical trials.

This article is part of the theme issue ‘Interplays between oxytocin and other neuromodulators in shaping complex social behaviours’.

## 1. Introduction

The ability to recognize and successfully interpret another person's emotions, emotion recognition, is essential in developing and maintaining social relationships, and poor emotion recognition is a risk factor for various psychiatric disorders [1]. Good emotion recognition therefore not only reduces the risk of developing poor mental health but also enables an individual to pursue a successful social life. The neuropeptide oxytocin (OT) has been found to play an important role in social cognition, and specifically, in improving emotion recognition [2–5]. This research has encouraged significant interest in the therapeutic potential of intranasal OT (IN-OT) as an intervention for individuals with emotion recognition difficulties [6]. Numerous randomized control trials have investigated the benefit of OT administration in various clinical populations, including autism spectrum disorder [7–19], schizophrenia [20–31], post-traumatic stress disorder [32,33], Prader-Willi syndrome [34], social anxiety disorder [35] and postnatal depression [36]. The success of these trials, however, has been mixed. One potential explanation for this, is that IN-OT has several caveats that limit its current usefulness.

Firstly, the pharmacokinetics of IN-OT administration are not fully understood. Consequently, it is not clear how many doses per day it would be necessary to prescribe to achieve the elevated OT concentrations that are associated with positive effects on social cognition. Based on current research [37–40], this may require several doses per day, which carries both cost and convenience considerations. Secondly, intranasal administration of OT is not straightforward. Indeed, researchers follow complicated guidelines [41] which would need to be imparted to patients to ensure correct administration. Thirdly, despite the enthusiasm to use IN-OT as a therapeutic intervention, relatively little research has investigated the long-term effects of administration, either pharmacokinetically or behaviourally [8,42,43]. Thus, there are a number of caveats to bear in mind when considering IN-OT as a therapeutic intervention.

Conversely, psychological interventions are typically cheaper and easier to disseminate. Several psychological interventions have been created to improve social interaction in autism [44,45] and schizophrenia [46,47]. Emotion recognition training, however, not only offers a discrete targeted intervention and measurable outcome but also the potential for wider positive effects on social behaviour. A detailed discussion regarding the potential of emotion training as an intervention is provided by Hunnikin & van Goozen [48]. One such intervention, the Cardiff Emotion Recognition Training (CERT) programme, has been found to not only increase emotion recognition but also improve longer term, objectively recorded social behaviour [49,50]. By contrast, the long-term effects of IN-OT administration are not fully understood. There is therefore a need to understand whether a psychological intervention, like CERT, is as effective at improving emotion recognition, compared to IN-OT.

There is, however, theoretical rationale to suggest that a combined intervention strategy incorporating IN-OT with a psychological intervention designed to improve social functioning, could provide additive benefits. The social salience hypothesis [51,52] of OT proposes that OT increases one's awareness of social cues in the environment. Therefore, presenting social cues under the influence of IN-OT may increase the salience of these cues, and therefore bolster the success of the intervention. Indeed, a handful of studies have investigated this theoretical understanding.^1^ The first such study administered IN-OT/placebo (twice daily) for six weeks to young adults with early psychosis with an additional dosage before each psychological intervention session (two hours each week, for 6 weeks), finding no additive effect of OT to the training [53]. Another study also investigated the additive effect of IN-OT (versus placebo) to social skills training in adults with schizophrenia, finding no evidence for this effect or an effect of training [54,55]. These studies, however, added a physiological intervention to a psychological intervention, and it was therefore not possible to ascertain whether IN-OT alone or training alone afforded the most social benefit. To our knowledge, only two studies to date have implemented this full (2 × 2) design, investigating whether a single administration of cognitive bias training and/or IN-OT in young children [56] and healthy students [57] improved feelings of maternal support or social imagery, respectively. Both studies, however, report null effects for IN-OT, but crucially also for all or some aspects of their psychological intervention, and it is, therefore, difficult to compare whether IN-OT or training had any/the most social benefit.

All four studies reported no additional benefit of IN-OT. One potential explanation for this is that the psychological interventions used were aimed at either large social constructs (i.e. social functioning) or social biases. Although the OT literature has suggested OT plays a diverse role in social behaviour, the most robust finding is that IN-OT increases emotion recognition [2–5]. A psychological intervention targeting emotion recognition specifically would therefore not only help focus the content of the psychological intervention but also focus on one of the most reliable IN-OT findings.

There are, however, several known moderators of the effect of IN-OT. First, OT effects are often emotion-specific. For example, a recent meta-analysis found that although there was an overall effect of OT on emotion recognition, this was driven by effects of OT on happy and fearful faces specifically [2]. Second, OT effects tend to be stronger for those with social difficulties [51]; indeed this is a strong driver for the therapeutic interest in OT. Relatedly, OT effects have also been shown to be moderated by task difficulty (for example by manipulating emotional intensity or stimulus duration) [2,51]. Thus, it will also be important for research to understand whether there are emotion-specific differences between IN-OT and CERT, and for whom these interventions may provide the biggest benefit.

Here we present a pilot, proof-of-concept study in which we compared IN-OT against a psychological intervention designed to improve emotion recognition, the CERT, in healthy volunteers. Firstly, we sought to compare the effect of the CERT *against* IN-OT (compared to their respective control conditions). Secondly, incorporating the social salience hypothesis, we hypothesized that the emotional cues presented during the CERT would become more salient to an individual who receive IN-OT before completing the training. It follows that if the cues presented during the training are more salient, one would anticipate that IN-OT would have a synergistic effect on the outcomes of the training, resulting in improved emotion recognition. In summary, we examined the effect of each intervention on emotion recognition: in isolation; in combination; and compared to their respective control conditions.

## 2. Methods

### (a) Participants and ethics

One hundred and four male undergraduates (*M*_age_ = 19.90, s.e.m. = 2.26) from Cardiff University took part in the study. The study aimed to recruit 120 participants. This value was determined based on previous OT administration studies in healthy populations [2,58]. As such, the study aimed to recruit more than double the standard sample size of previous studies, while acknowledging that the purpose of the study was to serve as pilot data for future research. Ultimately, 104 participants took part in the study, and no participants were lost across the two testing sessions. Data collection occurred January–November 2018. The study was approved by the School of Psychology Ethics Committee at Cardiff University (EC.17.03.14.4860R) and adhered to the Declaration of Helsinki. Participants from the School of Psychology were recruited via an online participation system; participants from other schools were recruited via an email advert. Participants were required to pass medical screening before taking part in the study, provided written informed consent prior to taking part and a signed statement of health before leaving the testing facility. Participants were not allowed to take part if they had a history of cardiovascular disease, neurological or mental health disorders. They were asked to avoid alcohol consumption 24 h prior to their session, and to avoid smoking and caffeine 2 h before. Female participants were excluded from taking part because (i) they would be required to take a pregnancy test prior to taking part, presenting additional ethical concerns; and (ii) there is some evidence that OT concentrations fluctuate during the menstrual cycle [59,60] and that this is affected by contraceptives [59]. Participants from the School of Psychology were awarded course credits in compensation for taking part; participants from other schools were paid for taking part.

### (b) Cardiff Emotion Recognition Training (CERT)

**Figure 1.**
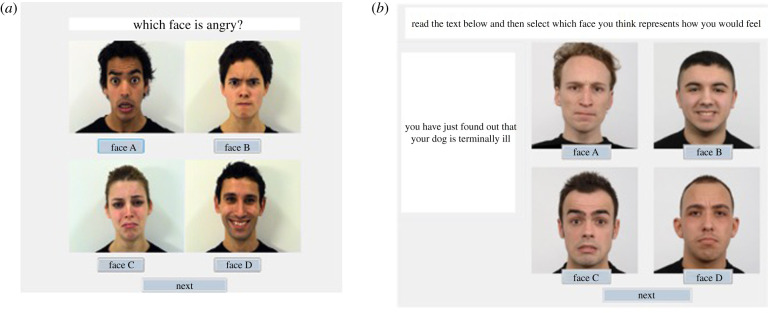
Illustrative examples of trials from the CERT including when participants had to select the correct facial expression (*a*) based on the emotion label shown and (*b*) on the emotional vignette.

In the present study, participants completed a shortened version of the CERT training [49,50] (https://emotionrecognition.cardiff.ac.uk/index.php) focusing on learning to recognize four basic emotions (happy, sad, fear and anger). In the current version of the training, participants begin with a specific introduction to the four different emotions, describing their similarities and differences using well-validated pre-existing stimuli [61]. Participants then completed different activities including being presented with four emotions and asked to click on the correct facial expression for the emotion label shown (figure 1*a*), being asked to compare facial expression for similar features, and matching emotional expressions to emotional vignettes (figure 1*b*).

### (c) Control training

A control training programme was designed to control for exposure to faces and attending to various facial features. In this way, any differences between the CERT and control training condition could be associated specifically with pertaining to emotional cues, as opposed to faces in general or the eye and mouth regions of the face. Participants were asked to identify eye colour, hair colour and gender of faces presented. Participants were therefore asked to look globally at the face to ascertain gender but also locally to focus on specific regions of the face, such as the eyes and hair. Faces were presented in three blocks, in an attempt to replicate the different activities and increasing difficulty presented in the CERT: in the first block the faces were presented upright; in the second block the faces were Thatcherized [62]; in the final block the faces were presented upside down [62]. The order of questions regarding eye/hair colour and gender were randomized across blocks.

### (d) Facial expression recognition task

A modified version of the facial expression recognition (FER) task from Hubble *et al*. [58] was used to assess participants' accuracy in identifying facial expressions of emotion. Participants saw three male and three female actors from the Ekman & Friesen [63] series, representing the same four basic emotions that participants were trained on during the CERT (happy, sad, fear and anger) and a neutral expression. Emotional expressions were presented at two different intensities (high and low; differing intensities were created by morphing each expression with the actors’ neutral face), for three different durations (200 ms, 300 ms and 600 ms).

We recruited healthy males who we expected to present with average emotion recognition ability, and as such would recognize easy items (those that were high intensity and presented for longer) at 80–90% accuracy. Therefore, careful consideration was given to create a range of difficulty during the task, in anticipation that effects from the interventions might be tested more accurately for harder items. Moreover, research suggests that 300 ms provides an interesting cut-off between implicit and explicit effects on IN-OT [2].

Participants saw a total of 162 faces: six times for each unique variable combination for emotional expressions, and once for each neutral face at all three durations (because intensity is not applicable to neutral faces). A trial consisted of a black screen for 500 ms, then a black screen with a white fixation cross for another 500 ms, then a face was presented (for either 200, 300 or 600 ms), immediately after the face disappeared, participants were asked to identify the emotion using a forced-choice paradigm. When participants had selected their answer, the next trial began, consequently there was no missing data for this task. The task was presented using Tobii Studio (https://www.tobii.com/).

Participants completed the FER twice (first at least two weeks before their experimental session to assess baseline emotion recognition accuracy). Two fixed, queso-random versions of the task were created in order to (i) ensure the participants saw the same faces but in a different order each time to avoid learning effects, and (ii) to ensure that participants did not, by chance, see a long sequence of one particular emotion. For each session, an average percentage recognition accuracy score was created for each emotion, intensity and duration. Finally, to examine the effect of the interventions, participants' baseline scores were subtracted from their experimental session scores. In this way, positive values indicate that participants identified more facial expressions correctly after the experimental session, negative values indicate that they identified fewer facial expressions, and 0 indicates that their emotion recognition was exactly the same.

### (e) Protocol

Participants took part in a randomized, double-blind, placebo-controlled, between-subjects study. On arrival, participants provided written informed consent and completed the Emotion Quotient (EQ; [64]), Autism Quotient-Short AQ (AQ-S; [65]), and the State and Trait Anxiety Index (STAI; [66]), before self-administering either a placebo (PL) nasal spray which was chemically matched to the OT spray apart from the active ingredient or 24 IU (three puffs of 4 IU per nostril) of OT. Sprays were administered in line with detailed guidelines [41] and under the supervision of the experimenter (KD; to eliminate inter-experimenter effects, KD conducted all experimental testing). Both nasal sprays were manufactured by St Mary's Pharmaceutical Unit, Cardiff (http://www.wales.nhs.uk/sites3/home.cfm?orgid=828), in line with a randomization list created by an independent researcher. Nasal spray administration was double-blind, bottles were numbered and given in sequential order. Participants were blind to training condition, but the experimenter was aware in order to load the correct training programme. Training condition was assigned on an alternating basis. Participants then completed the two-scale version of the Wechsler Abbreviated Scale of Intelligence [67] as a filler task for 30 min to ensure training was completed under peak OT concentrations [37–39]. At this time, participants completed either the CERT or the control training programme, which took approximately 15 min to complete. Immediately after the training, participants completed the FER (also taking approximately 15 min to complete). Participants completed a further 30 min’s worth of social cognition tasks before being fully debriefed at the end of the study.

### (f) Data analysis

A 2 (drug: OT/PL) × 2 (training: CERT/control) × 4 (emotion: happy/sad/anger/fear) × 3 (duration: 200/300/600) × 2 (intensity: high/low) mixed-model Analysis of Variance (ANOVA) was carried out, with the first two factors being between subjects, and the remaining factors being within subjects. Normality was assessed using skewness values and was within acceptable levels. For all analyses, where the assumption of sphericity was not met, Greenhouse-Geisser values are reported. Interaction effects were decomposed using simple effects analysis and all comparisons were Bonferroni corrected. The main effects and interactions regarding emotion, intensity and duration were in line with hypotheses and are not the primary focus of this paper; these are reported in the electronic supplementary material, S1. The data were analysed using SPSS 25 [68]. All data and analysis code for the present study are available on OSF (https://osf.io/cbfru/).

Finally, the questionnaire data for the EQ, AQ-S and STAI were averaged across their respective subscales and reliability analyses performed on the relevant subscales. For the purposes of this study, only the trait subscale of the STAI, social skills and mind reading of the AQ-S and overall EQ were chosen because we were only interested in trait assessments and did not anticipate a high prevalence of autistic traits assessed by the remaining subscales of the AQ-S. All subscales achieved good reliability scores (Cronbach's *α* ≥ 0.667), apart from the EQ (Cronbach's *α* = 0.505) which was not analysed further.

## 3. Results

First, to confirm that there were no differences in baseline emotion recognition across experimental groups, a separate univariate ANOVA was carried out. As expected, there was no significant difference in baseline FER performance across the four groups (*F*_3,100_ = 0.255, *p* = 0.857, $\eta_{p}^{2}=0.008$). Secondly, to confirm that there were no differences in social traits across experimental groups, a one-way ANOVA was carried out. As expected, there was no significant difference across the four groups for trait anxiety (*F*_3,100_ = 0.359, *p =* 0.782), mind reading (*F*_3,100_ = 0.615, *p =* 0.607) or social skills (*F*_3,100_ = 1.083, *p =* 0.360). Table 1 provides a summary of sample characteristics across the experimental groups.

**Table 1.** Sample size and descriptives (mean and standard deviation) for participants' self-reported anxiety, autistic-like traits and baseline facial emotion recognition accuracy across experimental conditions.

experimental condition	*n*	STAI-trait	AQ-mind reading	AQ-social skills	baseline FER (%)
placebo, control	26	44.0 (11.0)	2.9 (0.5)	2.9 (0.3)	82.8 (7.9)
placebo, training	28	44.9 (9.2)	3.0 (0.6)	2.8 (0.4)	82.7 (7.3)
oxytocin, control	24	42.3 (8.8)	3.0 (0.6)	3.0 (0.6)	82.8 (5.5)
oxytocin, training	26	42.8 (11.1)	3.1 (0.5)	3.0 (0.5)	81.4 (7.1)

**Figure 2.**
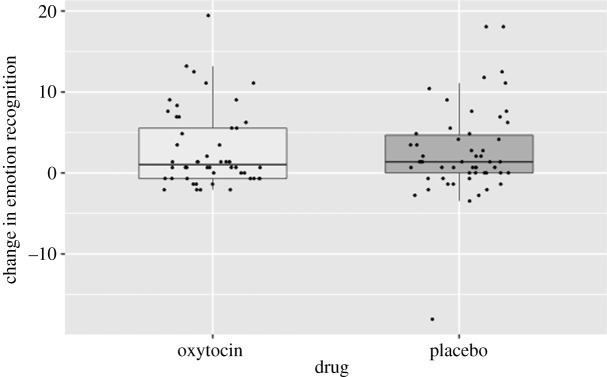
There was no effect of IN-OT on emotion recognition accuracy in the current study.

**Figure 3.**
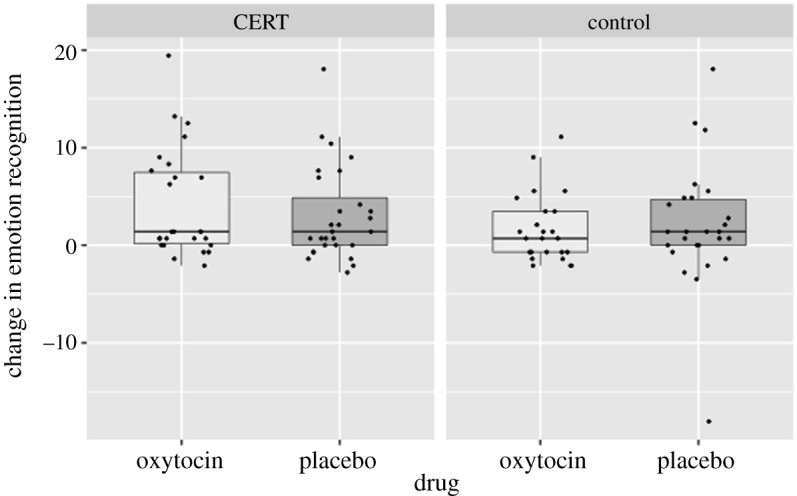
There was no significant additive effect of IN-OT in combination with the CERT.

The main model revealed that there was no main effect of training (*F*_1,100_ = 2.921, *p* = 0.091, $\eta_{p}^{2}=0.028$) or drug (*F*_1,100_ = 0.094, *p* = 0.759, $\eta_{p}^{2}=0.001$; figure 2) and no interaction (*F*_1,100_ = 0.466, *p* = 0.497, $\eta_{p}^{2}=0.005$; figure 3). However, there were several significant interactions with training.

There was a significant interaction between duration and training (*F*_1.87,187.20_ = 4.308, *p* = 0.017, $\eta_{p}^{2}=0.041$), such that, when collapsing across all emotions, participants who completed CERT identified more facial expressions correctly at 300 ms, compared to those who completed the control training (there were no differences for 200 ms or 600 ms). This lower order interaction was moderated by emotion in three-way interaction between emotion, duration and training (*F*_5.35,534.58_ = 2.182, *p* = 0.051, $\eta_{p}^{2}=0.024$). Participants who completed CERT specifically identified more angry facial expressions correctly, compared to those who completed the control training, when faces were presented for 200 ms *and* 300 ms (but not at 600 ms); and identified more sad facial expressions correctly, compared to those who completed the control training, when faces were presented for 300 ms (but not at 200 ms or 600 ms). There were no significant effects for happy or fearful expressions.

This interaction was further qualified by intensity (*F*_4.88,488.02_ = 2.161, *p* = 0.059, $\eta_{p}^{2}=0.021$), revealing that the previous findings were split by the emotional intensity of the facial expressions. Participants who completed CERT identified more *low intensity* angry expressions correctly but only when they were presented for 200 ms (and not at 300 ms or 600 ms) but identified more *high intensity* angry expressions correctly when presented for 300 ms (and not at 200 ms). Interestingly, they identified significantly *fewer* high intensity angry expressions correctly when presented at 600 ms. Moreover, the previous finding regarding sad facial expressions was also moderated by emotional intensity, such that participants who completed CERT specifically identified more *high intensity* sad expressions presented at 300 ms (and not at 200 ms or 600 ms, or any duration at low intensity). Finally, there were also significant effects on happy and fearful expressions, such that participants who completed CERT identified more *low intensity* happy expressions presented for 300 ms (and not 200 ms or 600 ms), and more *low intensity* fearful expressions presented for 600 ms (and not 200 ms or 300 ms).

Finally, there was a trend towards a significant four-way interaction between emotion, intensity, training and drug condition (*F*_3,300_ = 2.392, *p* = 0.069, $\eta_{p}^{2}=0.023$). Participants in the placebo condition who completed CERT identified more low intensity angry facial expressions correctly, compared to those who completed the control training (*F*_1,100_ = 7.655, *p* = 0.007, $\eta_{p}^{2}=0.071$). There was no corresponding improvement for those in the oxytocin condition (*F*_1,100_ = 0.034, *p* = 0.854, $\eta_{p}^{2}<0.001$). Those in the oxytocin condition who completed CERT did, however, identify more low intensity happy expressions correctly, compared to those who completed the control training (*F*_1,100_ = 3.895, *p* = 0.051, $\eta_{p}^{2}=0.037$). Again, there was no corresponding improvement for those in the placebo condition (*F*_1,100_ = 0.059, *p* = 0.808, $\eta_{p}^{2}=0.001$). It should be noted, however, that when simple effects analyses were carried out to examine direct differences between drug conditions, no significant results were found. Thus, strong caution is advised when reflecting on these findings.

There are two key findings from this analysis: that there was a consistent effect of CERT on recognizing angry expressions; and, as might be expected, the healthy males recruited to the pilot study had good emotion recognition ability. In order to understand what the possible effects of intervention might be for individuals with emotion recognition difficulty, we carried out an exploratory follow-up analysis based on these results: first, in order to avoid ceiling effects and following indications in the full model, we chose to focus on the most difficult faces (i.e. those presented for 200 ms at low intensity); second, due to the consistent finding in the full model, we chose to focus on angry expressions; third, we included participants' social traits as covariates. The resulting analysis was a 2 (drug: OT/PL) × 2 (training: CERT/placebo) univariate ANOVA with anxiety, mind reading and social skills as covariates, on participants responses to low intensity angry facial expressions presented at 200 ms.^2^

Replicating earlier findings, there was a main effect of training (*F*_1,97_ = 4.090, *p* = 0.046, $\eta_{p}^{2}=0.040$) such that participants who completed the CERT identified more angry expressions correctly (*M* = 10.436, s.e.m. = 3.795) compared to those who completed the control training (*M* = −0.669, s.e.m. = 3.944). There was no main effect of drug (*F*_1,97_ = 0.084, *p* = 0.772, $\eta_{p}^{2}=0.001$) and no interaction (*F*_1,97_ = 1.303, *p* = 0.256, $\eta_{p}^{2}=0.013$). All covariates were non-significant (anxiety: *F*_1,97_ = 0.652, *p* = 0.421, $\eta_{p}^{2}=0.007$; mind reading:, *F*_1,97_ = 0.017, *p* = 0.896, $\eta_{p}^{2}<0.001$; social skills:, *F*_1,97_ = 0.317, *p* = 0.575, $\eta_{p}^{2}=0.003$).

## 4. Discussion

We present a pilot study comparing the effect of IN-OT administration against a psychological intervention designed to improve emotion recognition, CERT, in healthy male volunteers to investigate whether a psychological intervention or IN-OT provided the most social benefit and whether a combined intervention strategy could provide additive benefit. There was no effect of IN-OT on emotion recognition (compared to PL), however, participants who completed the CERT identified more difficult angry facial expressions correctly (compared to those who completed a control training paradigm).

The finding that IN-OT did not improve emotion recognition contradicts several recent meta-analyses, which found that single administration studies of IN-OT had a reliable effect on emotion recognition [2–4]. As noted previously, however, our study recruited healthy volunteers with no known difficulties in emotion recognition, and results confirmed that participants were approaching ceiling effects for emotion recognition accuracy, despite incorporating both easy and difficult items into the outcome measure. Several studies have suggested that when incorporating moderating factors, the positive social effects of IN-OT are often only seen in individuals who experience social difficulties [33,54,69–71]. We conducted an exploratory analysis to investigate this possibility, however, our measures of social difficulty did not impact our findings. We note, however, that there was very little variation in these scores, particularly in the AQ-S subscales. Our pilot study should therefore be replicated in various clinical samples in order to clarify whether IN-OT or emotion recognition training provides the greatest emotion recognition improvement.

By contrast, the finding that CERT improved recognition of angry expressions consistently, and in certain conditions other negative expressions, replicates previous research [49,50] in which participants who completed three sessions of the CERT demonstrated improved anger recognition and general negative emotion recognition with reduced anti-social behaviour six months later. Emotion recognition training may therefore provide a specific and beneficial psychological intervention to support not only emotion recognition but also social interactions more widely.

There were numerous findings regarding the duration of stimuli presentation. This factor was specifically included based on a previous IN-OT meta-analysis [2] which found that while IN-OT had a small effect on improving overall emotion recognition, it had a moderate effect on improving emotion recognition for happy and angry facial expressions presented for less than 300 ms, and fearful facial expressions presented for greater than 300 ms, suggesting that emotion-specific effects of OT may vary as a function of stimulus presentation. Contrary to our predictions, we found no significant interactions between drug condition and duration, but several with training. It is interesting, therefore, that in general, our results demonstrate that CERT also tended to improve emotion recognition around this 300 ms cut-off, which Shahrestani and colleagues referred to as ‘early phase recognition’ reflecting implicit processing [72]. Specifically, our finding that CERT improved recognition of less than or equal to 300 ms angry facial expressions mirrors their IN-OT finding, which may explain why this effect was found for participants in the PL condition, and not for those in the OT condition. However, strong caution is advised when interpretating results from this interaction given the absence of any effect when comparing drug conditions directly. Thus, it would be interesting for future research to investigate whether CERT is indeed targeting, or seeing improvements as a result of increased ‘early phase recognition’.

By contrast to our hypothesis based on the social salience hypothesis of OT [51,52], our data do not support the notion of an additive effect of IN-OT to psychological interventions. This finding may be explained by potential ceiling effects for emotion recognition, preventing a main and interaction effect of IN-OT, or may reflect a genuine finding replicating all four previous studies that have examined the additive potential of IN-OT [53–57]. Regardless, several studies have now found no additive effect of IN-OT, yet there is continued interest in its therapeutic potential. It is important to reflect, therefore, on the caveats of IN-OT administration.

Firstly, the pharmacokinetics of OT administration are not fully understood. New research is still examining which administration technique and concentrations are optimal for elevated OT concentrations and associated positive effects on social cognition [40,73–75]. The most common dosage of IN-OT administration, 24 IU, is associated with increased OT concentrations for approximately 2 h, at which time concentrations are returning to baseline [37–40]. Importantly, this research implies that therapeutic interventions of IN-OT would require multiple doses per day to maintain OT concentrations associated with the desired effects. Assuming one would want the effects to last during daylight hours, and that elevated OT concentrations last for two hours, this could require as many as seven doses a day. It is therefore not surprising that many of the clinical trials cited in the introduction failed to find a beneficial social effect of IN-OT as the majority of trials included only one or two doses of IN-OT per day [7,8,13,14,16,20,22–30,33,34,36,42]. Multiple doses a day, however, would not only be inconvenient for patients but also carry potential cost implications. To address this issue, a promising longer lasting compound has recently been developed [76–78], in which beneficial behavioural effects were seen 16–24 h after administration, suggesting the compound has the potential to be delivered just once a day. These studies, however, were conducted in rodent models and the pharmacokinetics were not fully defined. The creation of OT superagonists provides exciting potential but extensive research is now required to understand whether the behavioural effects associated with previous OT administration studies are also apparent for these new compounds in humans, using different administration techniques. In the meantime, the existing literature using the traditional 24 IU of IN-OT remains the most relevant literature, and therefore longevity and cost remain relevant concerns.

Secondly, relatively little is known about the long-term effects of IN-OT administration. A handful of trials have documented the behavioural effects of administering IN-OT for longer than seven weeks [8,12,20,27,31,42,43], but only three assessed whether behaviour change was found during follow-up assessments after their interventions (ranging from 4 weeks to 1 year post intervention) [8,42,43]. Indeed, two of these studies were published just last year, highlighting the existing need to further understand the longevity of long-term IN-OT intervention. While more research is needed to understand the long-term behavioural effects, to our knowledge, no research has investigated the long-term pharmacokinetics. Whether, for example, individuals may become desensitized (and therefore require dose adjustments) over time is unknown.

Thirdly, basic research into the social effects of IN-OT is carried out under strict supervision. This is primarily to ensure consistency in self-administration protocols in order to avoid differences in the dosage achieved. Guastella and colleagues [41] published a long and detailed set of guidelines for researchers that would need to be imparted to patients if administration were to occur unsupervised (which is likely, assuming the need for multiple doses per day), if optimal doses are to be achieved.

Finally, the majority of IN-OT research to date has been carried out on male populations. This is often due to logistical constraints around administering IN-OT to women because there is some evidence that OT concentrations fluctuate over the menstrual cycle and with different contraceptives [60]. In addition, because OT is used to induce parturition, it can be necessary for female participants to complete a pregnancy test prior to taking part in a research study, which has additional cost and ethical implications. Indeed, the current study excluded females on this basis, and acknowledge this as an important limitation. Unfortunately, because the majority of research to date has been carried out on males it is unclear whether, or for what phenomena, sex differences are observed in behavioural effects of OT. There is therefore a need to include more mixed-sex samples in basic research to inform future clinical trials, particularly for disorders that specifically effect females (e.g. postnatal depression).

There are several further limitations in the current study that should be acknowledged. First, and related to the previous discussion, although recruiting a male-only sample enabled us to replicate previous research using CERT, it remains an unanswered question whether CERT can improve emotion recognition in females. Second, the current study did not investigate the longevity of these effects. Evidence suggests that the CERT is associated with beneficial behavioural effects six months after completion [49,50]. As previously discussed, the longevity of IN-OT's behavioural effects is not fully understood; however, research suggests that the beneficial social effects are not sustained beyond the time that OT concentrations are elevated. Consequently, combining IN-OT with the CERT may be mutually beneficial in that OT administration prior to the CERT may improve the effects associated with the training *and* the CERT may increase the longevity of effects associated with IN-OT. Future research is needed to understand whether combined interventions are associated with better longevity compared to single interventions. Third, the current study recruited healthy volunteers and, as expected, participants performed well on the task and demonstrated a limited range in social difficulties, thus limiting our ability to investigate the potential moderating role of social traits on IN-OT effects as has been seen previously (e.g. [51]). Fourth, the current study employed a visual based training programme. A recent study found that children with autism significantly improved their ability to interpret emotions from auditory stimuli after an auditory training intervention [79]. Investigating training programmes in different modalities may thus also prove fruitful for future research.

There are two specific strengths of the current study. First, the study recruited almost double the sample size (*n* = 104) to previous clinical studies [24,53,55] (and the same sample size to previous studies in healthy volunteers [56,57]) examining IN-OT and psychological interventions. Second, the study employed a full 2 × 2 design allowing us to directly compare whether participants who completed the CERT *or* received IN-OT displayed the greatest emotion recognition accuracy. Only two studies have used this approach [56,57], both published very recently (both also single administration trials in healthy volunteers), demonstrating the current need to understand whether psychological interventions by themselves can offer the same, or better, support to social functioning compared to IN-OT which has several draw backs. It is perhaps not a coincidence that both of these studies were also carried out in healthy volunteers. Clinical patients may be recruited via opportunity sampling if they are already participating in existing psychological interventions, however, further research in clinical samples using this full 2 × 2 design over a longer period of time is required to answer this important question.

In conclusion, we found no effect of IN-OT and a positive effect of an emotion training programme on healthy males' emotion recognition. Future directions for this research include investigating whether emotion recognition specifically, or social training in general, offers the same/better benefit to clinical populations compared to IN-OT, as has been argued here.

## Data Availability

All data and analysis code for the present study are available on OSF (https://osf.io/cbfru/). Electronic supplementary material is available online [80].

## Endnotes

1 Of note, only Cacciotti-Saija *et al*. [53] had been published when the current study was designed. The four remaining papers were published after data collection for the current study had finished.

2 All covariates were included in one model. Variance Inflation Factor scores were assessed for each covariate. All VIFs were <1.2, we therefore conclude there was no multicollinerity between covariates.
